# Internal validation of an 11-yr prediction model for new vertebral fractures using the vertebral bone quality score: a prospective cohort study

**DOI:** 10.1093/jbmrpl/ziaf155

**Published:** 2025-09-25

**Authors:** Takeru Yokota, Koji Otani, Yuji Endo, Kinshi Kato, Kenji Kobayashi, Takehiro Watanabe, Takuya Kameda, Yoichi Kaneuchi, Kazuyuki Watanabe, Takuya Nikaido, Miho Sekiguchi, Yoshihiro Matsumoto

**Affiliations:** Department of Orthopaedic Surgery, Fukushima Medical University School of Medicine, Fukushima 960-1295, Japan; Department of Orthopaedic Surgery, Fukushima Medical University School of Medicine, Fukushima 960-1295, Japan; Department of Orthopaedic Surgery, Fukushima Medical University School of Medicine, Fukushima 960-1295, Japan; Department of Orthopaedic Surgery, Fukushima Medical University School of Medicine, Fukushima 960-1295, Japan; Department of Orthopaedic Surgery, Fukushima Medical University School of Medicine, Fukushima 960-1295, Japan; Department of Orthopaedic Surgery, Fukushima Medical University School of Medicine, Fukushima 960-1295, Japan; Department of Orthopaedic Surgery, Fukushima Medical University School of Medicine, Fukushima 960-1295, Japan; Department of Orthopaedic Surgery, Fukushima Medical University School of Medicine, Fukushima 960-1295, Japan; Department of Orthopaedic Surgery, Fukushima Medical University School of Medicine, Fukushima 960-1295, Japan; Department of Orthopaedic Surgery, Fukushima Medical University School of Medicine, Fukushima 960-1295, Japan; Department of Orthopaedic Surgery, Fukushima Medical University School of Medicine, Fukushima 960-1295, Japan; Department of Orthopaedic Surgery, Fukushima Medical University School of Medicine, Fukushima 960-1295, Japan

**Keywords:** bone quality, vertebral bone quality score, magnetic resonance imaging, vertebral fracture, prediction model, internal validation, validation study, TRIPOD statement

## Abstract

The vertebral bone quality score is used to assess bone quality using magnetic resonance imaging. This study aimed to perform internal validation of a previously developed prediction model for the occurrence of new vertebral fractures (NVFs) over 11 yrs using the vertebral bone quality score. A prospective cohort of 157 participants from the Minami-Aizu study, with lumbar magnetic resonance imaging follow-ups over 11 yrs, was analyzed from an initial 200-participant cohort, applying the exclusion criteria. The primary outcome was the presence or absence of NVFs over 11 yrs. The predictors included age, sex, existing vertebral fractures at baseline, and VBQ scores. The prediction model, constructed using multiple logistic regression analysis and the Akaike information criterion, was evaluated for its discrimination power using the receiver operating characteristic curve and area under the curve (AUC). Internal validation was performed using the bootstrapping method with model reconstruction through regularization to correct for overfitting. In the multiple logistic regression analysis, predictors with a *p*-value of <.10 in multiple logistic regression analysis and <.05 in other analyses were considered statistically significant. New vertebral fractures occurred in 29 of the 157 participants. The AUC of the original model was 0.84 (95% confidence interval [CI]: 0.77-0.92). Bootstrapping revealed overfitting, which led to model reconstruction with regularization. The AUC of the regularized model was 0.84 (95% CI, 0.77-0.91), with no significant overfitting. The regularized model showed discrimination power equivalent to that of the original model without overfitting. A prediction model corrected for overfitting may be effective for long-term VF prediction. Future studies should investigate the external validity and clinical impact of the model.

## Graphical Abstract

**Graphical Abstract:**
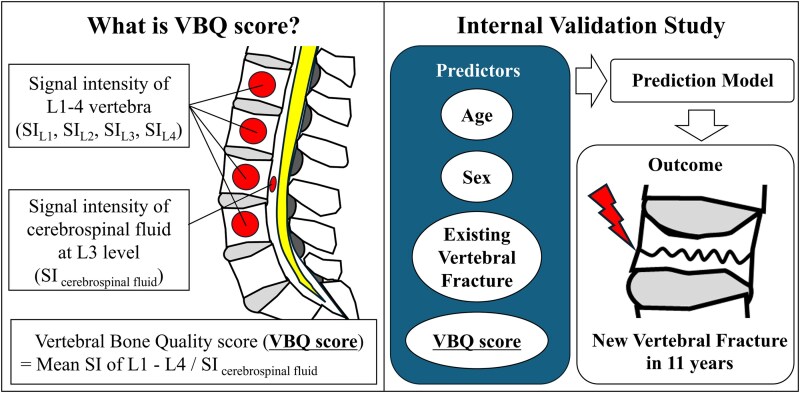

## Introduction

Osteoporosis is a disease characterized by decreased bone strength,^1^ which increases the risk of fractures. Bone strength depends on bone density and quality;^1^ however, methods for measuring bone quality remain underdeveloped. Recently, the vertebral bone quality (VBQ) score, which assesses bone quality using magnetic resonance imaging (MRI), has shown promising results.^2^^,^^3^ The VBQ score quantifies the degree of fat infiltration within the vertebrae using MRI T1-weighted images. Ehresman et al. suggested that the VBQ score has a superior predictive ability for new fractures compared to bone density alone.^3^

In our previous study,^4^ we developed a TRIPOD-compliant^5^ VBQ-based prediction model for new vertebral fractures (NVFs), incorporating VBQ score, age, sex, and existing vertebral fractures (EVFs). Although the model calculates fracture probability, it was susceptible to overfitting. We hypothesized that internal validation with overfitting correction would confirm its predictive accuracy, reliability, and applicability across diverse patient populations, despite a modest sample size. This study therefore aimed to validate the model internally to enhance its clinical utility and support improved decision-making in osteoporosis management.

## Materials and methods

The study was conducted in accordance with the Declaration of Helsinki and was approved by both the institution’s data protection officer and ethics committee (committee number: 17000061). All participants provided written informed consent for MRI and participation in the study.

This study was conducted in accordance with the TRIPOD statement,^5^ which outlines guidelines for validation studies of prediction models. The planning and execution of the study followed these guidelines to ensure rigorous validation.

### Study population and data collection

This study utilized data from the Minami-Aizu study, which included ~11 yrs of follow-up with 1862 participants (697 males and 1165 females) who underwent specific health checkups in 2004.

In Japan, specific health checkups are government-supported medical examinations aimed at early detection of metabolic syndrome and other health conditions. Among those who underwent the health checkups, 1862 individuals provided informed consent to participate in the Minami-Aizu study in 2004. The participants in this study were residents of Tadami-machi, Tateiwa-mura, and Ina-mura in Fukushima Prefecture, Japan, with ages ranging from 19 to 93 yrs.^4^^,^^6^

**Figure 1:**
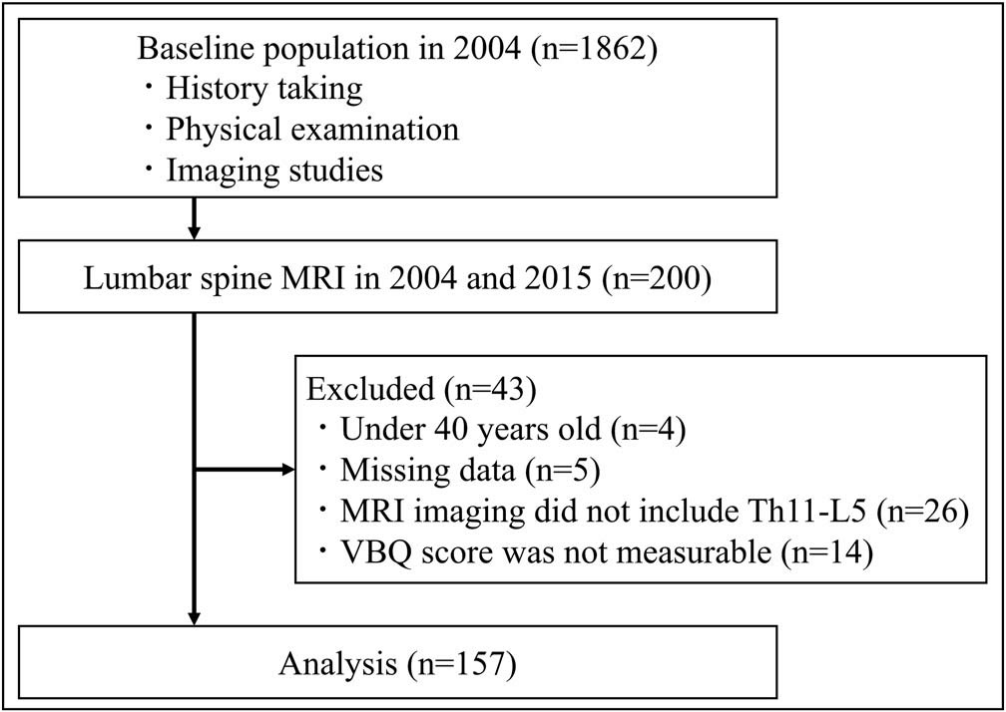
Flowchart of the study. MRI, magnetic resonance imaging.

Among the 1862 individuals, 459 underwent lumbar spine MRI in 2004, and 200 underwent follow-up MRI in 2015. These participants in both 2004 and 2015 included all individuals who voluntarily agreed to undergo MRI imaging. Exclusion criteria included participants under 40 yrs of age in 2004, those with missing data, inadequate imaging coverage from the 2004 MRI (Th11-L5), and those for whom the VBQ score could not be calculated from the 2004 MRI. A total of 43 participants were excluded: four for age, five for missing data, 26 for insufficient MRI coverage, and 14 for issues with VBQ score measurement, with some participants meeting multiple exclusion criteria. The final sample consisted of 157 participants (Figure 1).

This study employed updated selection and exclusion criteria compared to previous studies. A previous study adjusted for risk factors using MRI data and health checkup information, demonstrating the feasibility of constructing a prediction model solely based on MRI-derived data. This approach enabled the inclusion of cases previously categorized as having missing data.

### Magnetic resonance imaging

T1-weighted images were obtained using two MRI machines in 2004 and two MRI machines in 2015. These MRI machines used in 2004 for VBQ score measurement were described in detail (Table S1) in previous studies.^4^ All images were measured using a workstation (ZioCube, Mita, Minato-ku, Tokyo, Japan) at Fukushima Medical University (Fukushima City, Fukushima Prefecture, Japan).

### Assessment of NVF, VBQ score, and predictors

The predictors of NVFs were assessed in 2004 at the start of this study. These predictors included VBQ score, age, sex, and presence of EVFs. Detailed methods for measuring the outcomes and predictors are described below.

In this study, the primary outcome was the occurrence of NVF within 11 yrs of the year 2004. New vertebral fracture was assessed using lumbar spine Magnetic Resonance (MR) mid-sagittal images from 2004 to 2015. New vertebral fracture was defined as present if a reduction in vertebral height of 20% or more was observed in any part of the vertebral body (anterior, central, or posterior) within the T11 to L5 levels. The 20% threshold was independently determined based on previous reports.^7^^,^^8^ This method was also applied to individuals with EVF. Two investigators independently assessed the NVFs. If either investigator identified an NVF, the case was classified into the NVF group; otherwise, it was classified into the no NVF group. The diagnostic concordance between the two examiners was 89.3%, with a kappa coefficient of 0.574, indicating moderate agreement, as reported previously.^4^

**Figure 2:**
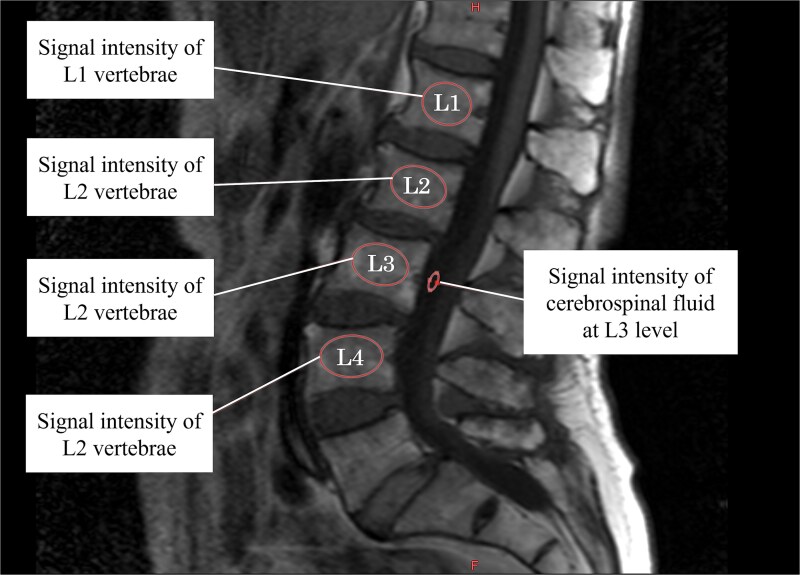
Measurement of the vertebral bone quality (VBQ) score. The VBQ score was measured on mid-sagittal T1-weighted images of lumbar spine magnetic resonance imaging (MRI). The VBQ score was calculated by dividing the average signal intensity within the vertebrae from L1 to L4 by the signal intensity of cerebrospinal fluid at the level of L3.

**Figure 3:**
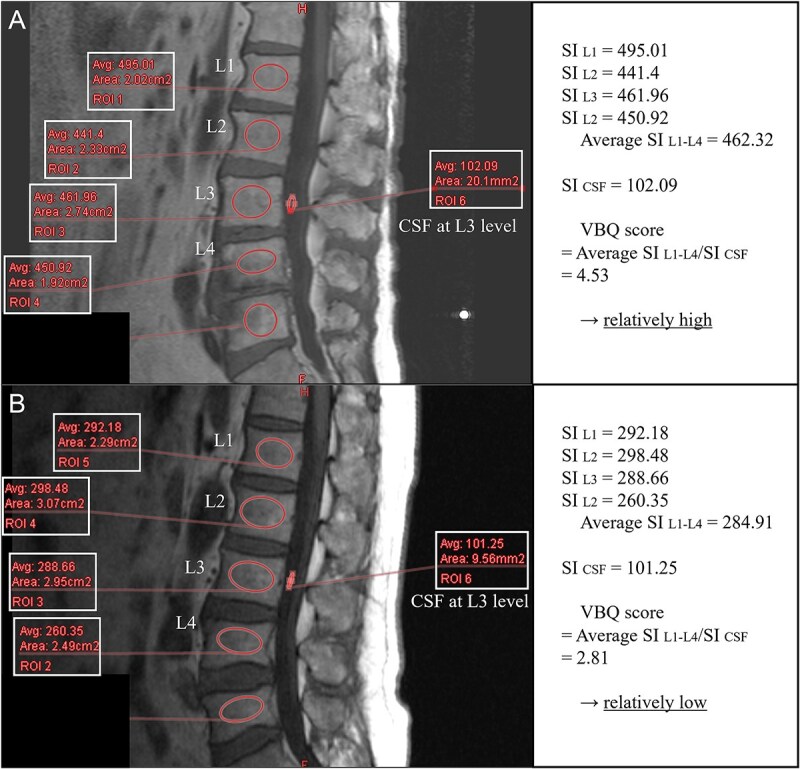
Example of vertebral bone quality (VBQ) score measurement. (A) An example with a relatively high VBQ score, suggesting poor bone quality. (B) An example with a relatively low VBQ score, indicating good bone quality. Avg, average; CSF, cerebrospinal fluid; L, lumbar, ROI, region of interest; SI, signal intensity; VBQ, vertebral bone quality.

First, the VBQ score in 2004 was calculated according to the report by Ehresman et al.’s report.^3^ In the MRI T1-weighted images, the intramedullary signal intensity in the lumbar spine from the first to fourth lumbar vertebrae (SI L1-4) and the signal intensity of the spinal fluid at the third lumbar spine level (SI fluid) were measured. The VBQ score was computed as the ratio of SI L1-4 to SI fluid (Figures 2 and 3). In individuals with EVF, SI measurements were taken, including the affected vertebral levels, and the VBQ score was calculated using the same method described above. The VBQ scores were independently measured by two investigators, and the intraclass correlation coefficient was calculated to evaluate the reliability of the VBQ score measurements. The average VBQ score from 2004 was used for the analysis.

Age and sex information were recorded from MRI data in 2004. The presence of EVF was evaluated based on the findings from the lumbar spine MR mid-sagittal images. Existing vertebral fractures were diagnosed using a semiquantitative method. If a vertebra in the Th11 to L5 range showed a reduction in vertebral height of ≥20%, it was determined to have EVF.^8^

### Predictor selection and model development

Multiple logistic regression analysis was used to identify potential predictors of NVFs, including VBQ score, age, sex, and the presence of EVFs. Predictor selection was further refined using the Akaike information criterion (AIC).^9^ The final predictors were used to construct the prediction model (original model), which estimated the probability of vertebral fractures (VFs) occurring within 11 yrs.

### Internal validation of the prediction model

The performance of the original model was assessed in terms of discrimination power and calibration.

The discrimination power was evaluated using the area under the curve (AUC) of the receiver operating characteristic (ROC) curve. Additionally, to assess the contribution of the VBQ score to the improvement in the predictive model's discrimination ability, the ROC curve and AUC for the prediction model excluding the VBQ score as a predictor were also presented. To statistically evaluate the contribution of the VBQ score, a DeLong test was used to compare the AUCs of the models with and without the VBQ score.

The calibration was assessed by examining the slope and intercept of the calibration plot.

The internal validity of the original model was verified using bootstrapping to detect overfitting. If overfitting was identified, the prediction model was reconstructed using regularization to address this issue. The corrected model (regularized model) was then reevaluated for discrimination power, calibration, and internal validity.

### Evaluation of clinical utility

The clinical utility of the final prediction model (original or regularized model) was evaluated by calculating the sensitivity, specificity, positive predictive value (PPV), negative predictive value (NPV), false positive rate (FPR), and false negative rate (FNR) at a cutoff probability of 15%. The suitability of this cutoff value was assessed using a net benefit analysis.

### Sample size

This study utilized existing samples and information; therefore, no pre-study sample size calculations were performed.

### Missing data

Participants with missing data were excluded from analysis.

### Statistical analysis

#### Participant characteristics

*T*-tests were performed for normally distributed continuous variables. The Wilcoxon signed-rank test was used for nonnormally distributed continuous variables. Chi-square or Fisher's exact test was used for categorical variables.

#### Predictor selection and model development

Multiple logistic regression analyses evaluated the VBQ score, age, sex, and the presence of EVFs as predictors of NVFs. VBQ score and age were treated as continuous variables, whereas sex and EVF were treated as categorical variables. After the initial logistic regression, predictor selection was refined using AIC.^9^ Predictors with an AIC lower than the criterion were excluded.

#### Model performance and internal validation

The original model was constructed on the basis of the beta coefficients of the selected predictors. The discrimination power of the model was evaluated using the AUC of the ROC curve. The calibration was assessed using the slope and intercept of the calibration plots.

Bootstrapping with 200 resamples was used to calculate the optimism-adjusted slope. An adjusted slope of 0.9 or higher indicated sufficient internal validity, whereas a slope below 0.9 suggested overfitting. In cases of overfitting, the model was reconstructed using least absolute shrinkage and selection operator (LASSO) regression,^10^^,^^11^ which enhances model interpretability and prediction accuracy.

#### Decision curve analysis

To assess the clinical usefulness of the model, decision curve analysis was performed by calculating the net benefit^13^ across a range of threshold probabilities (0.05-0.30). A net benefit greater than zero indicated that the model provided a heightened clinical utility compared to the "treat all" or "treat none" strategies.

For the main analysis, the net benefit was calculated using the following formula:


\begin{eqnarray*} && \mathrm{Net}\ \mathrm{Benefit}=\left(\mathrm{True}\ \mathrm{Positives}/\mathrm{Total}\ \mathrm{Sample}\right)\\&&\quad -\, \left(\mathrm{False}\ \mathrm{Positives}/\mathrm{Total}\ \mathrm{Sample}\right)\\&&\quad \times\, \left(1-\mathrm{Cutoff}\ \mathrm{Probability}\right)\kern-2pt{/}\mathrm{Cutoff}\ \mathrm{Probability}. \end{eqnarray*}

This conventional approach estimates the clinical value of identifying true positives while penalizing false positives.

In a supplementary analysis aimed at minimizing false negatives, clinical utility was defined as the number of interventions avoided per 100 patients:


\begin{align*} & \mathrm{Interventions}\ \mathrm{avoided}\ \mathrm{per}\ 100\ \mathrm{patients}= \\& \quad 100\times \left[ \left(\mathrm{True}\ \mathrm{Negatives}/\mathrm{Total}\ \mathrm{Sample}\right)\right.\\& \quad -\, \left(\mathrm{False}\ \mathrm{Negatives}/\mathrm{Total}\ \mathrm{Sample}\right)\\&\left. \quad \times\, \left(1-\mathrm{Cutoff}\ \mathrm{Probability}\right)\kern-2pt/\mathrm{Cutoff}\ \mathrm{Probability}\right]. \end{align*}

This formulation highlights the model's effectiveness in reducing undertreatment by prioritizing correct identification of true negatives.

#### Clinical utility

The clinical utility of the final prediction model was evaluated using a cutoff probability of 15%. This cutoff was based on the fracture risk assessment tool (FRAX) threshold.^12^ A cross table was created to calculate the model's sensitivity, specificity, PPV, NPV, FPR, and FNR.

For multiple logistic regression analysis, a *p*-value of <.1 was considered statistically significant, whereas a *p*-value of <.05 was used for other analyses. All statistical analyses were performed using R version 4.3.1.

## Results

### Outcomes

New vertebral fractures occurred in 29 (18.5%) of 157 participants.

### Patient characteristics

The mean age of all participants was 63.9 yrs (standard deviation [SD]: 8.4), with 114 (72.6%) females. Existing vertebral fractures were observed in 38 participants (24.2%). The mean VBQ score was 4.01 (0.81).

In the no NVF group, the mean age was 62.7 yrs (SD: 8.4), with 87 (68.0%) females and 25 (19.5%) participants having EVF. Their mean VBQ score was 3.92 (0.81).

In the NVF group, the mean age was higher at 69.0 yrs (SD: 6.1), with a greater proportion of females (27 participants, 93.1%) and a higher incidence of EVF (13 participants, 44.8%). They also had a higher mean VBQ score of 4.41 (0.69).

Statistically significant differences were observed between the two groups across all predictors examined (Table 1).

**Table 1:** Patient characteristics.

	**Total *n* = 157**	**No NVF group *n* = 128**	**NVF group *n* = 29**	***p* Value** ^**a**^
**Age, mean (standard deviation)**	63.9 (8.4)	62.7 (8.4)	69.0 (6.1)	<.001
**Sex, female, *n* (%)**	114 (72.6)	87 (68.0)	27 (93.1)	.012
**EVF, *n* (%)**	38 (24.2)	25 (19.5)	13 (44.8)	.008
**VBQ score, mean (standard deviation)**	4.01 (0.81)	3.92 (0.81)	4.41 (0.69)	.003

Abbreviations: EVF, existing vertebral fracture, NVF, new vertebral fracture; VBQ score, vertebral bone quality score.

a *T*-tests, chi-square tests, or Fisher's exact tests were used for statistical analysis.

### Selection of predictors

**Figure 4:**
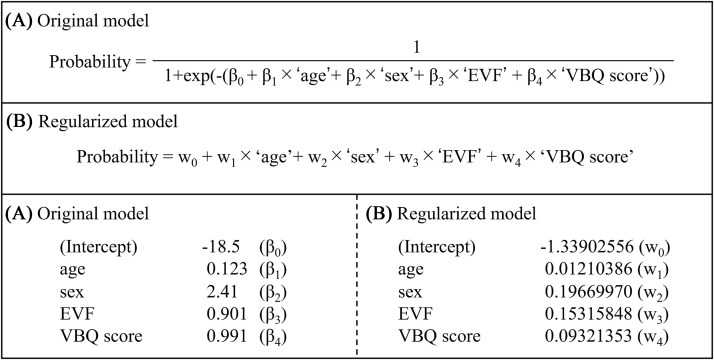
(A) Original model: a prediction model developed using multiple logistic regression analysis and the Akaike information criterion. (B) Regularized model: a prediction model created by applying a least absolute shrinkage and selection operator (LASSO) regression to the original model. Here, *β* represents the beta coefficients, and *w* refers to the weights. EVF, existing vertebral fractures; VBQ, vertebral bone quality.

Multiple logistic regression analysis identified age, sex, EVF, and VBQ score as significant predictors (Table 2). In addition, the AIC for these predictors exceeded the baseline value of 120.2 (Table 3). Therefore, these predictors were used to construct the original model (Figure 4A).

**Table 2:** Results of multiple logistic regression analysis.

	***β* Coefficients**	**Standard error**	***z* Value**	***p* Value**	
**(Intercept)**	−18.5	3.87	−4.77	1.84e-06	^***^
**Age**	0.123	0.038	3.24	.001	^**^
**Sex**	2.41	0.86	2.81	.005	^**^
**EVF**	0.901	0.523	1.73	.084	·
**VBQ score**	0.991	0.321	3.09	.002	^**^

Significant codes: “^***^” 0.001, “^**^” 0.01, “^*^” 0.05, “·” 0.1. Abbreviations: EVF, existing vertebral fracture; VBQ score, vertebral bone quality score.

**Table 3:** Comparison of AIC for predictors.

	**Df**	**Deviance**	**AIC**
**(None)**		110.2	120.22
**Age**	1	123.61	131.61
**Sex**	1	122.64	130.64
**EVF**	1	113.16	121.16
**VBQ score**	1	120.07	128.07

AIC, Akaike information criterion; Df, degrees of freedom; EVF, existing vertebral fracture; VBQ score, vertebral bone quality score.

### Internal validation

**Figure 5:**
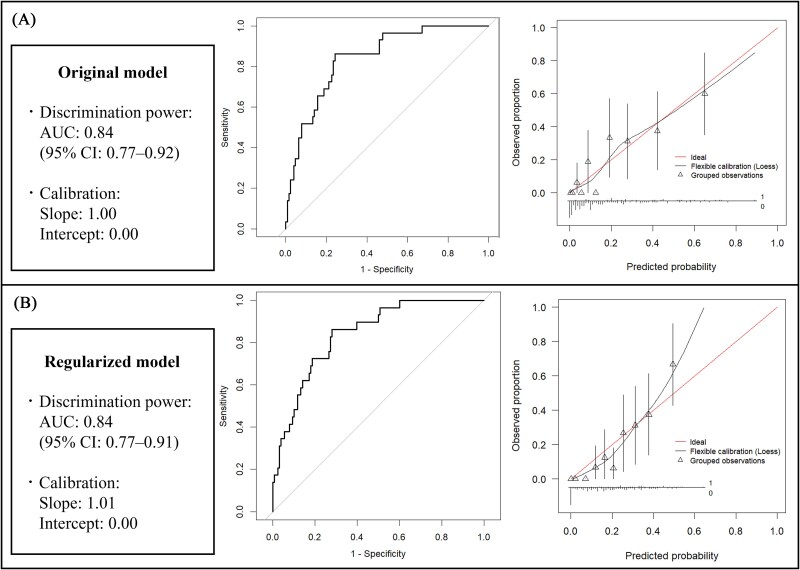
The receiver operating characteristic (ROC) curves and calibration plots for the prediction models: (A) original model and (B) regularized model. Discrimination power was assessed by calculating the area under the ROC curve. Calibration was evaluated using the calibration slope and intercept. AUC, area under the curve; CI, confidence interval.

The original model had an AUC of 0.843 (95% confidence interval [CI]: 0.771-0.916), with a calibration slope of 1.00 and an intercept of 0.00 (Figure 5A). Bootstrapping revealed an optimism-corrected slope of 0.864 (<0.9), indicating overfitting (Table 4). To correct for overfitting, a regularized model was constructed (Figure 4B).

**Table 4:** The results of bootstrapping for original and regularized models.

	**Index**	**Uncorrected**	**Training**	**Test**	**Optimism**	**Corrected**
**Original model**	Dxy	0.6864	0.7075	0.6758	0.0318	0.6546
	Intercept	0.0000	0.0000	−0.1360	0.1360	−0.1360
	Slope	1.0000	1.0000	0.8643	0.1357	0.8643
**Regularized model**	Dxy	0.6800	0.6794	0.6800	−0.0005	0.6805
	Intercept	0.0000	0.0000	0.0160	−0.0160	0.0160
	Slope	1.0000	1.0000	1.0042	−0.0042	1.0042

This table presents the results of bootstrapping performed with 200 resamples for both the original and regularized models. Definitions of the terms used are as follows: uncorrected: estimates from the original model; training: estimates based on the training dataset; test: estimates based on the test dataset; optimism: optimism correction using bootstrap methods (adjustment for overfitting); corrected: estimates after optimism correction; Dxy: a measure of the model's discrimination ability.

**Figure 6:**
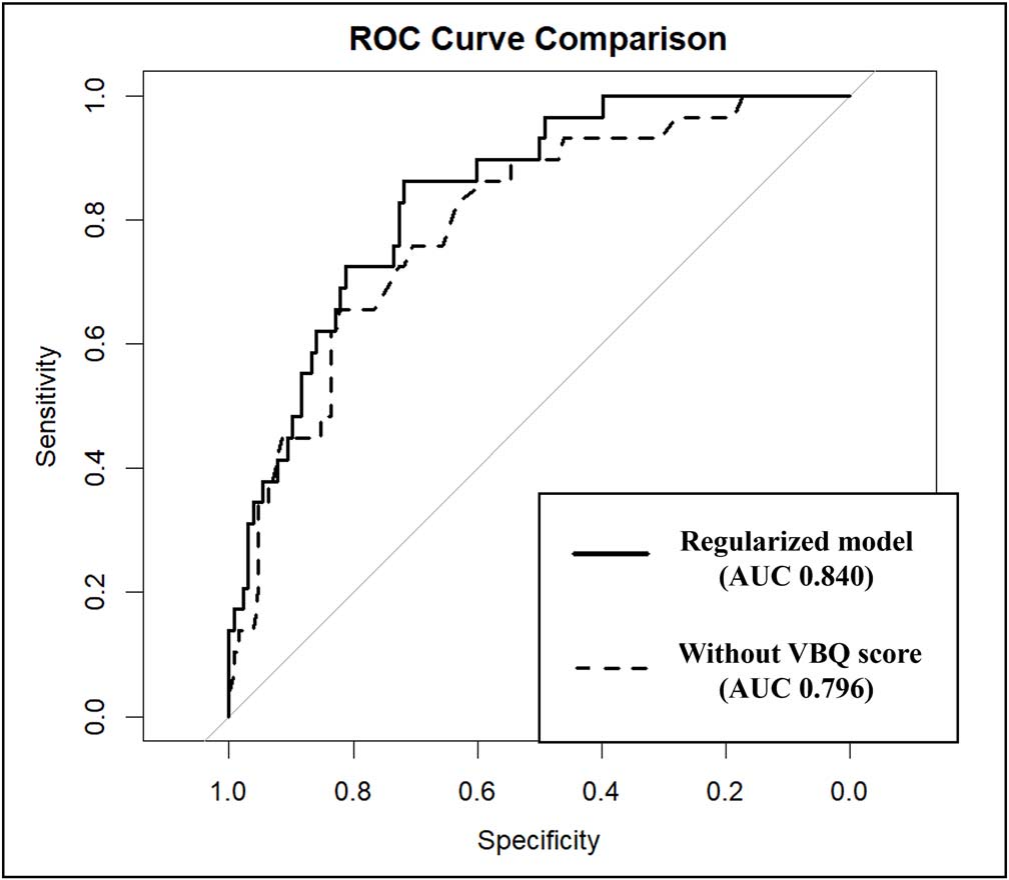
Comparison of ROC curves and AUC values. The solid line represents the ROC curve of the regularized model, while the dashed line represents the ROC curve of the model excluding the VBQ score. Both ROC curves and their corresponding AUC values are presented. AUC, area under the curve; ROC, receiver operating characteristic; VBQ, vertebral bone quality score.

The regularized model maintained an AUC of 0.840 (95% CI, 0.768-0.912), showing consistent discrimination power compared to the original model. The AUC of the model constructed by excluding the VBQ score from the predictors was 0.796 (95% CI, 0.710-0.883) (Figure 6), indicating that the inclusion of the VBQ score improved the AUC of the regularized model. However, this improvement did not meet the statistical significance threshold (*p* = .052), and no significant difference was observed. The calibration slope of the regularized model was 1.01, with an intercept of 0.00, indicating good calibration (Figure 5B).

Similarly, bootstrapping showed an optimism-corrected slope of 1.00, suggesting no overfitting of the regularized model (Table 4).

### Decision curve analysis

**Figure 7:**
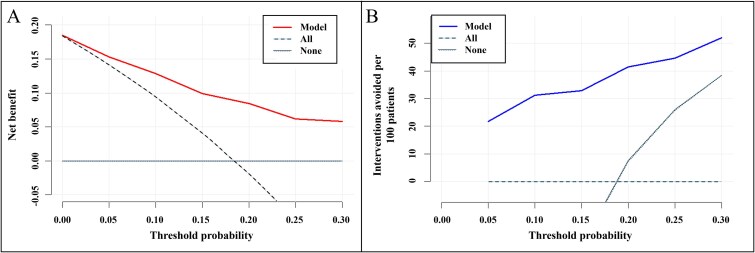
Decision curve analysis (DCA). DCA was performed across threshold probabilities from 0.05 to 0.30. (A) Conventional DCA plot showing the model’s net benefit (solid line) compared to the “treat all” (dashed line) and “treat none” (dotted line) strategies in reducing overtreatment by focusing on identifying true positives. (B) Clinical utility, defined as interventions avoided per 100 patients, for the model (solid line) versus “treat all” (dashed line) and “treat none” (dotted line) strategies. Higher values indicate fewer unnecessary interventions via correct identification of true negatives. Overall, the model consistently outperformed both default strategies across the range of thresholds.

Figure 7A and B show the model’s clinical utility across threshold probabilities (0.05-0.30). In Figure 7A, net benefit consistently exceeded both “treat all” and “treat none” strategies at every threshold, reflecting the traditional decision-curve framework that prioritizes true positives to reduce overtreatment.

In Figure 7B, clinical utility was defined as the number of interventions avoided per 100 patients, focusing on minimizing undertreatment by maximizing true negatives and minimizing false negatives. The model demonstrated positive clinical utility across thresholds, highlighting its robustness in reducing undertreatment.

At the clinically relevant threshold of 0.15, commonly used in FRAX-based risk assessment, the net benefits were 0.0992 in Figure 7A and 0.3291 in Figure 7B. Peak net benefit occurred at thresholds of 0.05-0.10 in Figure 7A and 0.25-0.30 in Figure 7B.

### Clinical utility

A diagram illustrating the cross table of the adjusted model showed the results for all 157 participants: 27 true positives, 65 false positives, 63 true negatives, and two false negatives. The sensitivity was 93.1%, specificity was 49.2%, PPV was 29.3%, NPV was 96.9%, FPR was 50.8%, and FNR was 6.9%.

## Discussion

In this study, we assessed the internal validity of a prediction model for NVFs using predictors such as age, sex, EVF, and the VBQ score. This validation enhances our understanding of the reliability and clinical applicability of this model.

Accurate prediction models are essential for effective VF management and decision making. Although our previous study developed a long-term prediction model for VFs, its performance raised concerns when applied to new or diverse patient populations.

In this study, although the original model demonstrated good discrimination power and calibration, it suffered from overfitting, which affected its internal validation. To address this, we applied LASSO regression to create a regularized model that maintained its strong discrimination power and calibration without overfitting. This internal validation step was crucial for ensuring the reliability and accuracy of the model. By addressing overfitting, we improved the generalizability of the model, making it a more reliable and consistent tool for clinical use.

In this study, we evaluated the clinical utility of using a 15% cutoff for predicted probabilities, consistent with the FRAX.^12^ This cutoff demonstrated high sensitivity and a low FNR, making it effective for screening the future risk of VF. However, our data did not fully capture the information necessary to calculate the 10-yrs incidence of new fractures using FRAX. Future research should compare the performance of established osteoporosis screening tools, such as FRAX, with our prediction model to further validate its efficacy.

Decision curve analysis showed that the model yielded greater net benefit than that provided by both “treat all” and “treat none” strategies across all thresholds (Figure 7A). In Figure 7B, clinical utility was defined as the number of interventions avoided per 100 patients, focusing on minimizing undertreatment by prioritizing true negatives and reducing false negatives. The model consistently demonstrated positive clinical utility across threshold probabilities, peaking at 32.9 interventions avoided per 100 patients at thresholds of 0.25-0.30. These findings highlight the model’s robustness and effectiveness in supporting clinical decision making under different strategies, extending beyond the conventional 15% cutoff. Future research should identify threshold ranges that optimally balance sensitivity, specificity, and net benefit to improve fracture risk stratification.

The VBQ score was developed to reflect intramedullary adiposity by capitalizing on the high signal intensity of bone marrow fat on MRI T1-weighted images.^14^ However, MRI signal intensity can vary significantly owing to different imaging conditions,^15^ potentially leading to substantial errors. To minimize such errors, the VBQ score calculation uses the signal intensity of the cerebrospinal fluid as a calibration reference.^3^

Moreover, the VBQ score predicts fracture incidence independently of bone mineral density. Ehresman et al. showed a relationship between VBQ score and new fracture incidence in a retrospective observational study with an average follow-up of 12.5 yrs.^2^ Compared to other bone quality indicators, such as the trabecular bone score and Hounsfield units,^16^ the VBQ score provides high fracture prediction accuracy without radiation exposure. Our study also confirmed the effectiveness of the VBQ score in predicting NVFs.

Relying solely on the VBQ score to predict fractures presents challenges. A DeLong test comparing model AUCs with and without VBQ revealed a difference of 0.044 (95% CI, −0.008 to 0.095; *p* = .052), narrowly missing the .05 significance threshold. Although adding VBQ trended toward better NVF discrimination, the improvement was not statistically significant. To assess its independent prognostic value, we examined VBQ alone, stratified by EVF status. Overall, its AUC for predicting NVF was 0.711 (95% CI, 0.605-0.817; *p* < .001; Table S2). Stratified analyses yielded an AUC of 0.753 (95% CI, 0.635-0.871; *p* = .001) in patients without EVF and 0.674 (95% CI, 0.476-0.872; *p* = .082) in those with EVF (Table S3). These results suggest VBQ predicts NVF more effectively in the absence of EVF but has limited utility when EVF is present. Age and sex also significantly influence NVF risk, often more than EVF, highlighting the need for a multifactorial prediction model.

Consistent with clinical observations, aging, particularly in females, was associated with increased fracture risk.^17^ Moreover, EVFs were a significant predictor, aligning with reports that patients with EVFs face a high risk of recurrent VFs.^18^^,^^19^ Previous studies have noted that patients with EVFs may not always exhibit high VBQ scores,^20^ possibly due to remodeling or mineralization at the fracture site. We hypothesize that severe vertebral collapse can render VBQ assessment infeasible. Consequently, we excluded participants whose VBQ scores were unobtainable due to complete vertebral collapse, ensuring that NVF evaluation was restricted to vertebrae with measurable baseline structures. While this exclusion maintained measurement reliability, it limits the model’s applicability to relatively healthy community residents or those with less severe EVFs.

We also compared VBQ scores between participants with and without EVFs to assess the influence of prevalent fractures on our model’s predictions (Table S4). The mean VBQ score in the EVF group (*n* = 38; 3.94 ± 0.75) was slightly lower than in the non-EVF group (*n* = 119; 4.03 ± 0.83; *p* = .61, *t*-test), and a box plot (Figure S1) confirmed the absence of extreme outliers. These findings suggest that the VBQ score remains relatively independent of prevalent fractures in measurable vertebrae, as the small difference is unlikely to have driven the overall model predictions.

This study has some limitations. First, its retrospective design may introduce selection bias: participants voluntarily attended health screenings in the Minami-Aizu region, potentially excluding those who experienced significant health declines, functional impairments, or relocation before the 2015 follow-up. Second, the small sample size (*n* = 157) and high loss to follow-up further limit the model’s generalizability. Consequently, our prediction model may apply primarily to relatively healthy community residents.

Second, as the study focused on community residents, the model’s applicability to individuals at high risk of complications, such as those in hospitals, remains unclear. The effectiveness of this model in predicting conditions specific to high-risk populations, such as diabetes, remains unclear. Further validation is required to assess the applicability of this model in these settings.

Finally, since this study utilized an existing dataset, we were unable to investigate fractures outside the vertebral bodies, bone mineral density (BMD), or the history of osteoporosis treatment. In particular, future external validation and performance comparisons will likely need to include models that incorporate BMD.

## Conclusion

In summary, this study validated the internal validity of a prediction model for NVFs using the VBQ scores. The performance of the model, corrected for overfitting through normalization, was satisfactory. Future research should focus on evaluating the external validity and clinical implications of this model.

## Supplementary Material

Supplementary_Figure_S1_ziaf155

Supplementary_Table_S1_ziaf155

Supplementary_Table_S2_ziaf155

Supplementary_Table_S3_ziaf155

Supplementary_Table_S4_ziaf155

## Data Availability

Data supporting the findings of this study are available upon reasonable request, subject to ethical and legal considerations.

## Permission to reproduce material from other sources

No materials from other sources were reproduced in this study.
