# Supplementary figures and images for: Internal validation of an 11-yr prediction model for new vertebral fractures using the vertebral bone quality score: a prospective cohort study
Source: JBMR Plus. 2025 Sep 25;9(11):ziaf155. doi: 10.1093/jbmrpl/ziaf155 (PMC12515476; doi:10.1093/jbmrpl/ziaf155)

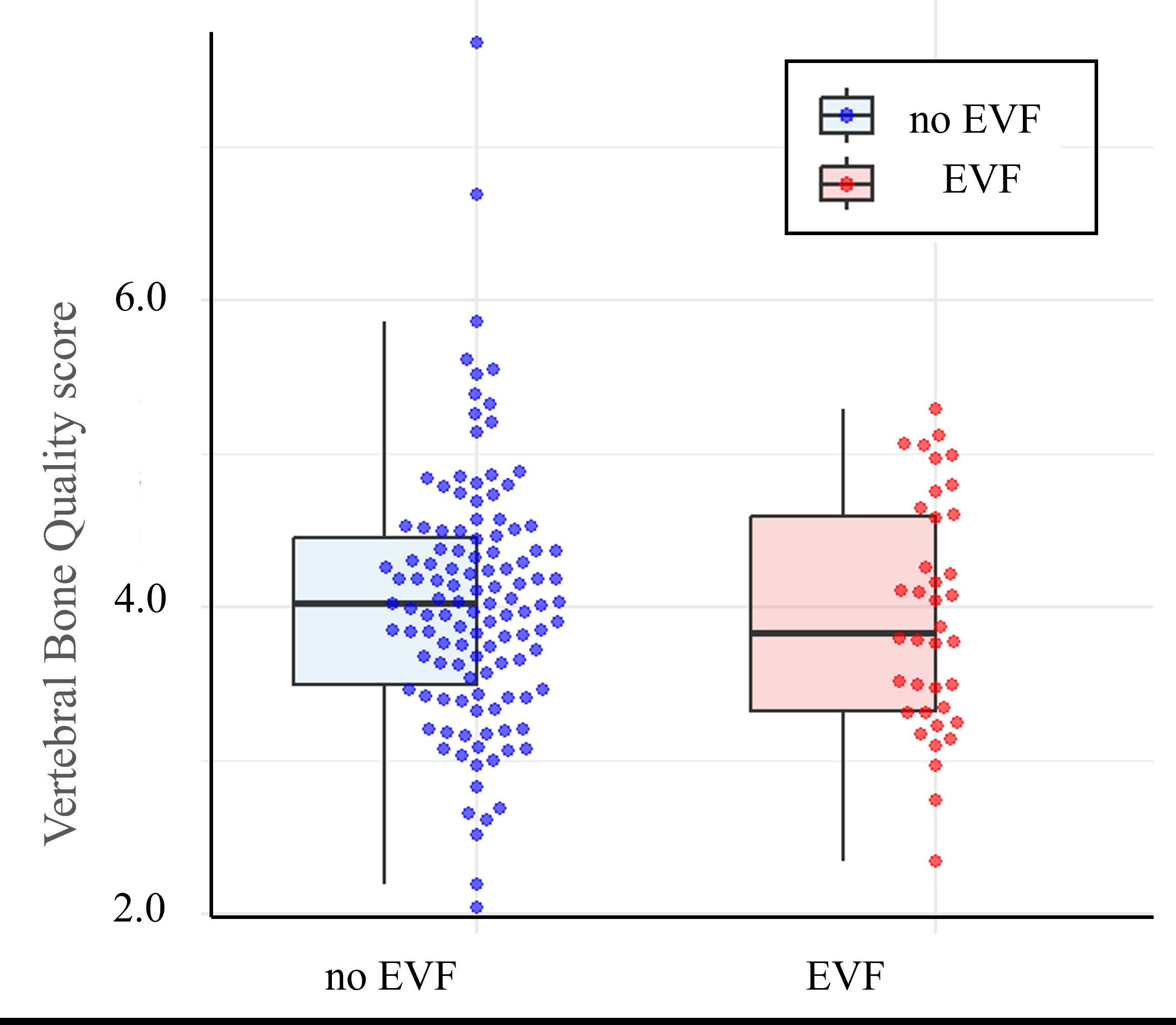

Supplement: Supplementary_Figure_S1_ziaf155 [file supplementary_figure_s1_ziaf155.jpeg]
