# Supplementary material for: Internal validation of an 11-yr prediction model for new vertebral fractures using the vertebral bone quality score: a prospective cohort study
Source: JBMR Plus. 2025 Sep 25;9(11):ziaf155. doi: 10.1093/jbmrpl/ziaf155 (PMC12515476; doi:10.1093/jbmrpl/ziaf155)
Supplement: Supplementary_Table_S1_ziaf155 [file supplementary_table_s1_ziaf155.docx]

Supplementary Table S1. Imaging characteristics and technical parameters for MRI scans used in vertebral bone quality score measurements

| Parameter | Toshiba EXCELART/P2 Pianissimo | Philips Gyroscan Intera Power |
| --- | --- | --- |
| Magnetic Field Strength (T) | 1.5 | 1.0 |
| Repetition Time (ms) | 4,000 | 4,500 |
| Echo Time (ms) | 108 | 120 |
| Slice Thickness (mm) | 5 | 5 |
| Inter-Slice Gap (mm) | 1 | 0.5 |
| Manufacturer | Toshiba | Philips |

Notes:
This table reports technical parameters for MRI scans conducted in 2004 to measure vertebral bone quality (VBQ) scores. Data are adapted from a prior publication by a member of our research team, with permission obtained from the publishing journal.
