# Supplementary material for: Internal validation of an 11-yr prediction model for new vertebral fractures using the vertebral bone quality score: a prospective cohort study
Source: JBMR Plus. 2025 Sep 25;9(11):ziaf155. doi: 10.1093/jbmrpl/ziaf155 (PMC12515476; doi:10.1093/jbmrpl/ziaf155)
Supplement: Supplementary_Table_S2_ziaf155 [file supplementary_table_s2_ziaf155.docx]

Supplemental Table S2: AUROC of VBQ score for predicting NVF (overall analysis)

|  | **AUC** | **Standard Error** | **p-value** | **95% Confidence Interval** | |
| --- | --- | --- | --- | --- | --- |
|  |  |  |  | Lower Bound | Upper Bound |
| VBQ score | 0.711 | 0.054 | 0.000 | 0.605 | 0.817 |

Notes: The test result variable Vertebral Bone Quality (VBQ) score has at least one tie between the positive and negative actual state groups, which may introduce bias in the statistics. Abbreviations: AUROC, area under the receiver operating characteristic curve; AUC, area under the curve; VBQ, Vertebral Bone Quality; NVF, new vertebral fracture.
