# Supplementary material for: Internal validation of an 11-yr prediction model for new vertebral fractures using the vertebral bone quality score: a prospective cohort study
Source: JBMR Plus. 2025 Sep 25;9(11):ziaf155. doi: 10.1093/jbmrpl/ziaf155 (PMC12515476; doi:10.1093/jbmrpl/ziaf155)
Supplement: Supplementary_Table_S3_ziaf155 [file supplementary_table_s3_ziaf155.docx]

Supplementary Table S3: AUROC of VBQ score for predicting NVF (stratified by EVF status)

| **Group** | **AUC** | **Standard Error** | **p-value** | **95% Confidence Interval** | |
| --- | --- | --- | --- | --- | --- |
|  |  |  |  | Lower Bound | Upper Bound |
| Patients without EVF | 0.753 | 0.060 | 0.001 | 0.635 | 0.871 |
| Patients with EVF | 0.674 | 0.101 | 0.082 | 0.476 | 0.872 |

Notes: The test result variable Vertebral Bone Quality (VBQ) score has at least one tie between the positive and negative actual state groups, which may introduce bias in the statistics. Standard errors were not reported.
Abbreviations: AUROC, area under the receiver operating characteristic curve; AUC, area under the curve; VBQ, Vertebral Bone Quality; NVF, new vertebral fracture; EVF, existing vertebral fracture.
