# Supplementary material for: Internal validation of an 11-yr prediction model for new vertebral fractures using the vertebral bone quality score: a prospective cohort study
Source: JBMR Plus. 2025 Sep 25;9(11):ziaf155. doi: 10.1093/jbmrpl/ziaf155 (PMC12515476; doi:10.1093/jbmrpl/ziaf155)
Supplement: Supplementary_Table_S4_ziaf155 [file supplementary_table_s4_ziaf155.docx]

Supplementary Table S4. Comparison of VBQ scores between participants with and without EVF

| **Group** | **N** | **Mean VBQ Score ± SD** | **Standard Error of Mean** |
| --- | --- | --- | --- |
| No EVF | 119 | 4.03 ± 0.83 | 0.076 |
| EVF | 38 | 3.94 ± 0.75 | 0.122 |

Note: Vertebral Bone Quality (VBQ) scores were calculated only for vertebrae with measurable structures, as severe Existing Vertebral Fractures (EVFs) (e.g., completely collapsed vertebrae) were excluded due to infeasible measurements.
